# Genome Sequence of the Banana Plant Growth-Promoting Rhizobacterium *Pseudomonas fluorescens* PS006

**DOI:** 10.1128/genomeA.00329-16

**Published:** 2016-05-05

**Authors:** Rocío M. Gamez, Fernando Rodríguez, Sandra Ramírez, Yolanda Gómez, Richa Agarwala, David Landsman, Leonardo Mariño-Ramírez

**Affiliations:** a Corporación Colombiana de Investigación Agropecuaria (CORPOICA), Centro de Investigación Caribia, Magdalena, Colombia; b Corporación Colombiana de Investigación Agropecuaria (CORPOICA), Centro de Investigación Tibaitatá, Cundinamarca, Colombia; c National Center for Biotechnology Information, National Library of Medicine, National Institutes of Health, Bethesda, Maryland, USA

## Abstract

*Pseudomonas fluorescens* is a well-known plant growth-promoting rhizobacterium (PGPR). We report here the first whole-genome sequence of PGPR *P. fluorescens* evaluated in Colombian banana plants. The genome sequences contains genes involved in plant growth and defense, including bacteriocins, 1-aminocyclopropane-1-carboxylic acid (ACC) deaminase, and genes that provide resistance to toxic compounds.

## GENOME ANNOUNCEMENT

Plant growth-promoting rhizobacteria (PGPR) are soil bacteria that may exist on the rhizosphere or colonizing plant roots and benefit them by providing growth promotion (1). *Pseudomonas* is one of the prominent PGPR genera that has been reported to stimulate growth in plants, such as cucumber, peppermint, blackberry, and banana (2–4). Bacterial metabolic pathways, including phosphate solubilization, 1-aminocyclopropane-1-carboxylic acid (ACC) deaminase activity, siderophores, and inole acetic acid (IAA) production, are well-known PGPR traits in *Pseudomonas fluorescens* (5). Root colonization by *P. fluorescens* is critical for the manifestation of their beneficial effects (6).

*P. fluorescens* PS006 was isolated from *Physalis peruviana* (7, 8) roots in Boyacá, Colombia, and its PGPR properties were evaluated in tomato and banana plants. Genomic DNA was extracted from bacterial overnight cultures using the UltraClean microbial DNA isolation kit (Mo Bio, Carlsbad, CA, USA), modified with additional mechanical cell disruption using the MicroBead solution. Genomic DNA was prepared for Illumina sequencing using Agilent SureSelect QXT libraries. The whole-genome sequencing was performed using Illumina Hi ScanSQ, generating 3,615,480 paired-end reads of 300 bp in length. The *P. fluorescens* PS006 genome was assembled using a reference-guided assembler, ARGO, developed at NCBI, and a *de-novo* assembler, SPAdes (9), resulting in an assembly with a total of 6,250,362 bp, a G+C content of 60.3%, and a genome coverage of 100.0×. Genome assemblies yielded 47 contigs, with *N*_50_ contig size of 257,296 bp. The contigs were annotated using the NCBI Prokaryotic Genome Automatic Annotation Pipeline (PGAAP) and were deposited at GenBank. The genome of *P. fluorescens* PS006 annotation revealed 5,543 genes, 5,474 coding sequences (CDSs), 4 rRNAs, 61 tRNAs, 4 noncoding RNAs (ncRNAs), and 61 pseudogenes.

The automatic annotation was enriched using RAST version 2.0 (10). We found in the genome of *P. fluorescens* PS006 a number of genes involved in the synthesis of 14 cofactors, with 25 genes in potassium metabolism, 52 genes for nitrogen metabolism, and 78 genes for phosphorus metabolism. Additionally, the genome contains genes previously described for health and plant defense, as follows: 14 genes for bacteriocins and ribosomally synthesized antibacterial peptides, 123 genes for resistance to antibiotics and toxic compounds, 12 genes for invasion and intracellular resistance, and 193 genes for environmental stress response. All these are genes that might be involved in plant growth promotion and are probably related to the interactions between the rhizobacteria and its host *Musa acuminata* (banana Williams), suggesting diverse pathways potentially involved in banana plant growth. Here, we announce the first genome sequence of a banana PGPR *P. fluorescens* strain in Colombia.

### Nucleotide sequence accession numbers.

This whole-genome shotgun project has been deposited at DDBJ/EMBL/GenBank under the accession no. LRMR00000000. The version described in this paper is version LRMR01000000. The BioProject accession no. is PRJNA236098.
